# *Cryptococcus neoformans* Ex Vivo Capsule Size Is Associated With Intracranial Pressure and Host Immune Response in HIV-associated Cryptococcal Meningitis

**DOI:** 10.1093/infdis/jit435

**Published:** 2013-08-14

**Authors:** Emma J. Robertson, Grace Najjuka, Melissa A. Rolfes, Andrew Akampurira, Neena Jain, Janani Anantharanjit, Maximilian von Hohenberg, Manlio Tassieri, Allan Carlsson, David B. Meya, Thomas S. Harrison, Bettina C. Fries, David R. Boulware, Tihana Bicanic

**Affiliations:** 1 Department of Infection and Immunity, St George's University of London, London, United Kingdom; 2 Infectious Disease Institute, Makerere University, Kampala, Uganda; 3 Division of Infectious Diseases & International Medicine, Department of Medicine, University of Minnesota, Minneapolis; 4 Department of Medicine, Albert Einstein College of Medicine, Bronx, New York; 5 Division of Biomedical Engineering, School of Engineering, University of Glasgow, United Kingdom

**Keywords:** *Cryptococcus neoformans*, cryptococcal meningitis, HIV, polysaccharide capsule, intracranial pressure, immune response, CSF, human

## Abstract

***Background.*** The *Cryptococcus neoformans* polysaccharide capsule is a well-characterized virulence factor with immunomodulatory properties. The organism and/or shed capsule is postulated to raise intracranial pressure (ICP) in cryptococcal meningitis (CM) by mechanical obstruction of cerebrospinal fluid (CSF) outflow. Little is known regarding capsule phenotype in human cryptococcosis. We investigated the relationship of ex vivo CSF capsular phenotype with ICP and CSF immune response, as well as in vitro phenotype.

***Methods.*** In total, 134 human immunodeficiency virus (HIV)-infected Ugandan adults with CM had serial lumbar punctures with measurement of CSF opening pressures, quantitative cultures, ex vivo capsule size and shedding, viscosity, and CSF cytokines; 108 had complete data. Induced capsular size and shedding were measured in vitro for 48 *C. neoformans* isolates.

***Results.*** Cryptococcal strains producing larger ex vivo capsules in the baseline (pretreatment) CSF correlated with higher ICP (*P* = .02), slower rate of fungal clearance (*P* = .02), and paucity of CSF inflammation, including decreased CSF white blood cell (WBC) count (*P* < .001), interleukin (IL)-4 (*P* = .02), IL-6 (*P* = .01), IL-7 (*P* = .04), IL-8 (*P* = .03), and interferon γ (*P* = .03). CSF capsule shedding did not correlate with ICP. On multivariable analysis, capsule size remained independently associated with ICP. Ex vivo capsular size and shedding did not correlate with that of the same isolates grown in vitro.

***Conclusions.*** Cryptococcal capsule size ex vivo is an important contributor to virulence in human cryptococcal meningitis.

*Cryptococcus neoformans*, an opportunistic fungus causing infection in immunocompromised hosts, is the most common cause of adult meningitis in Sub-Saharan Africa [1, 2] and a major cause of infectious mortality [3]. A distinctive diagnostic feature of *C. neoformans* in cerebrospinal fluid (CSF), as well as its most important virulence factor [4], is its polysaccharide capsule. The capsule is composed of branching polysaccharide fibers of glucuronoxylomannan (GXM, 90%–95% of capsular mass), and galactoxylomannan (GalXM) [5], whose highly dynamic biophysical and chemical structure confers phenotypic diversity, facilitating immune evasion and survival within the host [6].

During mammalian infection, the capsule undergoes dramatic changes in size, structure, and proportion relative to total cell volume, stimulated by factors such as iron limitation and physiologic CO_2_ [7, 8]. Shed GXM polysaccharide, commonly known as cryptococcal antigen (CrAg), has a large molecular weight and is viscous in solution [9], which may contribute to increased intracranial pressure (ICP), especially in the setting of a large fungal burden [10], by mechanically obstructing CSF outflow through arachnoid villi [11].

Many studies demonstrating the importance of the *C. neoformans* capsule in virulence have used laboratory strains in animal models. In an immunocompetent murine model, acapsular mutants were avirulent [12, 13]. In one murine study using clinical isolates from AIDS patients, capsule size within mouse brains correlated with virulence [14]. The capsule and actively shed GXM have deleterious effects on the immune response, including inhibiting phagocytosis, T-cell proliferation, and proinflammatory cytokine production [6]. A mucoid phenotypic switch variant (MC) of a serotype D strain produces larger capsules, associated with greater tissue shedding of more viscous GXM compared to the smooth variant (SM). MC elicits a more vigorous yet ineffective inflammatory response, exhibiting enhanced virulence in mice [15], and showed greater propensity than SM to cause raised ICP in a rat model [16].

The cryptococcal capsule has not been well characterized in vivo in human infection. Our study's objective was to test the hypothesis that diversity in the *C. neoformans* capsular phenotype in human cryptococcal meningitis (CM) is associated with the propensity to develop raised ICP and capacity to elicit a CSF inflammatory response. Given in vitro capsular phenotype is often assumed to represent in vivo phenotype, our second objective was to correlate ex vivo phenotype in human CSF with in vitro phenotype of corresponding clinical isolates grown in culture.

Using serial CSF samples and clinical isolates collected and examined prospectively in an African clinical trial cohort, we present the first study to describe the relationship between ex vivo *C neoformans* capsular phenotype and clinical and immune parameters in the human host.

## METHODS

### Study Population

Specimens were collected from a prospective cohort at Mulago Hospital, Kampala, Uganda, as a nested sub-study of the Cryptococcal Optimal ART Timing (COAT) trial (clinicaltrials.gov: NCT01075152). Inclusion criteria were human immunodeficiency virus (HIV)-infected, ART-naive persons ≥18 years presenting with a first CM episode confirmed by CSF culture and/or CrAg testing. Written informed consent was provided. Participants had serial lumbar punctures (LPs) on days 1, 7, and 14 of treatment with amphotericin B deoxycholate (0.7–1.0 mg/kg/day) and fluconazole (800 mg/day). CSF opening pressure (OP) was measured at each LP, up to 55 cm H_2_O. The trial had approval from the Research Ethics Committees of Makerere University, University of Minnesota, Mulago Hospital, and Uganda National Council for Science and Technology.

### Sample Collection and Storage

CSF supernatants and cryptococcal isolates (taken as a sweep across multiple colonies) were cryopreserved at −80°C prior to shipment to St. George's University, London (SGUL). *C. neoformans* were routinely grown at 37°C on either Sabouraud dextrose agar (SDA), or in Sabouraud dextrose broth (SDB) with shaking at 150 rpm, unless otherwise specified.

### Whole CSF Microscopy and Quantitative Cultures

Quantitative cultures were performed by serial 10-fold dilution (up to 1:10^5^), and 100 μL of each dilution inoculated onto SDA. Plates were incubated at 30°C for ≤14 days, and colonies counted at the lowest dilution showing discernible colonies, multiplying by the dilution to give colony-forming units (CFU) per mL CSF. Rate of clearance of infection, or early fungicidal activity (EFA), was determined by subject-specific linear regression of log_10_-transformed serial cultures by day of cryptococcal therapy, as described elsewhere [17, 18].

### CSF Supernatant CrAg and Cytokine Analysis

Whole CSF was centrifuged for 10 minutes at 3500 g, supernatant stored and shipped at −80°C to Minnesota. CrAg titers were determined using the CrAg Lateral Flow Assay (Immy, Oklahoma) semi-quantitatively, by performance of serial 2-fold dilutions of CSF, starting at 1:250. CSF cytokines were measured via Luminex (Bio-Rad, Hercules, CA) for interleukin 1β, interleukin 2, interleukin 4 (IL-4), interleukin 5, interleukin 6 (IL-6), interleukin 7 (IL-7), interleukin 8 (IL-8), interleukin 10, interleukin 12, interleukin 13, interleukin 17, granulocyte colony-stimulating factor, granulocyte macrophage colony-stimulating factor, interferon γ (IFN-γ), macrophage chemoattractant protein 1, macrophage inflammatory protein 1β , tumor necrosis factor α (TNF-α), and vascular endothelial growth factor (VEGF).

### Capsule Size Measurements

For ex vivo analysis fresh CSF was counter-stained with India ink, and capsule thickness ([total diameter - cell body diameter]/2), hereafter referred to as capsule size, measured within 1 hour of LP, using a 40× objective and an eyepiece grid micrometer (Wirsam Scientific, Cape Town). Digital photography via microscope C-mount documented views for external quality assurance.

For in vitro analysis, *C. neoformans* isolates were grown overnight in SDB. The following day 20 μL of culture was inoculated into 5 mL capsule-inducing media (Dulbecco's Modified Eagle Medium with 1% NCTC, 10% heat-inactivated fetal bovine serum) and incubated at 37°C with 10% CO_2_ for 48 hours. Cells were harvested by centrifugation at 950 × g for 5 minutes, counter-stained with India ink, imaged using 40× objective, and measured using ImageJ software (v1.44o). Mean capsular parameters were measured for 30–50 cells ex vivo and 50 cells in vitro.

### Cryptococcal Capsule Glucuronoxylomannan (GXM) Shedding in In Vitro Culture Supernatants and Ex Vivo CSF

*Cryptococcus neoformans* isolates were inoculated into 10 mL SDB, grown at 37°C until optical density (OD)_600_ was 0.7–1.1 and centrifuged twice at 950 g for 5 minutes. Supernatants were maintained at 4°C short-term or −80°C long-term. GXM concentration was determined on in vitro culture supernatants and corresponding patient CSF supernatants using the CrAg EIA (IMMY, Oklahoma). Briefly, 50 μL of specimen was added to 96-well plates coated with anti-GXM monoclonal antibody. Using specimen diluent, whole CSF was diluted 1:10, and culture supernatants diluted 1:100 and titrated using 1:5 dilutions. A positive control of 10 ng/mL cryptococcal serotype A GXM was included (all isolates tested were confirmed serotype A by polymerase chain reaction). EIA titers were calculated using the highest 450 nm absorbance value falling within the acceptable range (0.265–2.55). Final GXM concentration was determined by comparison of OD_450_ against the positive control. For comparison of in vitro-in vivo shedding, in vitro titers were normalized to OD_600_ of 1, and CSF titers normalized to a CSF fungal burden of 1 log_10_CFU/mL.

### GXM Production and Purification

GXM purification was performed as described elsewhere [19]. Briefly *C. neoformans* isolates were inoculated into 10 mL SDB and incubated overnight at 30°C. Cultures were added to 500 mL minimal media (10 mM MgSO_4_, 29.3 mM KH_2_PO_4_, 13 mM glycine, 3 μM thiamine-HCl, pH 5.5 with 15 mM glucose), and incubated for 11 days at 30°C with shaking. Cultures were centrifuged at 4000 g for 15 minutes (at 4°C), supernatant removed, and centrifuged again at 15 000 g for 15 minutes (4°C). Supernatant was passed through a 0.22 μM Millipore vacuum filter and concentrated using an Amicon filter (100 kDa cut-off). The GXM “jelly” was removed from the membrane and lyophilized in preparation for viscosity measurements.

### Viscosity Measurements

Viscoelastic properties of CSF supernatants and GXM solutions were measured using 2 microrheology techniques: passive video particle tracking and optical tweezers. The first was performed using an upright BH2 microscope (Olympus, Melville, New York) equipped with a Prosilica GV640M complementary metal-oxide semiconductor camera and 100× objective. Carboxylic-acid-modified polystyrene spheres (1 μm) were used as probes. Images were stored as AVI files (25 frames/sec). An average 30 particles per frame were tracked simultaneously in 10 minute movies, which were processed using a particle-tracking MATLAB code (University of Glasgow). CSF supernatant viscosity was measured by mean square displacement 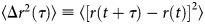 of the probes' trajectories *r*(*t*), using the formula:
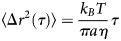

where *k_B_T* is thermal energy, *τ* is lag-time (or time interval), *a* is particle radius, and *η* fluid viscosity.

Microrheology measurements with optical tweezers were performed on purified GXM dissolved in ultrapure H_2_O (concentration range 10^−3^ mg/mL–10 mg/mL). Viscoelastic properties of GXM solutions were measured using both the analytical method and the optical tweezers setup described in Tassieri et al [20]. The linear viscoelastic properties of the solutions can be represented by the frequency-dependent complex shear modulus 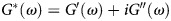, a complex number providing information on both the elastic (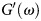) and viscous (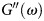) nature of the fluid. The complex modulus can be expressed as a function of the normalized position autocorrelation function 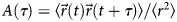 [21]:
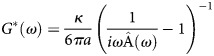

where 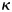 is trap stiffness and 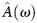 is the Fourier transform of 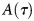. For this experiment, the viscosities of GXM solutions were evaluated as the value of 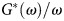 for 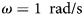.

### Statistical Analysis

Data were analyzed using SAS version 9.3 (SAS Institute, North Carolina) and Stata v11 (Statacorp, Texas). Correlations were assessed with Spearman rank correlation (ρ), categorical comparisons used the χ^2^ test, and continuous used the Kruskal-Wallis test. Linear regression was used to estimate the relationship of capsule size with EFA and CSF opening pressure. All CSF parameters significantly associated with CSF OP on univariate linear regression analysis (*P* ≤ .05), were combined into a multivariable model to investigate factors independently associated with ICP. Despite lack of significant univariate association in this study, baseline CSF fungal burden was also included in the multivariable model due to its previously described association with ICP [10]. Changes in cell body and capsule size during antifungal therapy were assessed using mixed effects modeling with a random term accounting for inter-person correlation over time.

## RESULTS

Among 134 HIV-infected persons with CM, 362 serial LPs were performed. At the baseline diagnostic LP, prior to antifungal therapy, 124 persons had CSF OP recorded and 122 persons had capsular parameters measured in real-time on fresh CSF. Complete data were available for 108 persons for capsule size, LP OP, and CSF cytokines. Table 1 shows baseline clinical variables and CSF parameters. Clinical parameters were characteristic of HIV-infected persons presenting with CM in Africa: median age 35 years, median CD4 count 16 cells/μL, high CSF fungal burden (median 5.1 log_10_ CFU/mL), and minimal CSF inflammation (median 10 lymphocytes/μL CSF).

**Table 1.** Baseline Clinical Variables and CSF Parameters at Diagnosis

Baseline Variable	*n*	Median (IQR)
Males, no. (%)	71 (53%)	…
Age, years	134	35 (30–40)
CD4 cells/μL	96	16 (6.5–44.5)
Altered mental status, no. (%)	39 (30%)	…
CSF parameters at initial diagnosis		
Opening pressure, cm H_2_O	124	28.5 (19.6–38.7)
Polymorphs /μL	126	0 (0–5)
Lymphocytes /μL	126	10 (<5–49)
Quantitative *C. neoformans* culture (log_10_ CFU/mL)	130	5.1 (4.32–5.61)
Cryptococcal Antigen LFA Titer, 1:x	119	8000 (2000–16 000)
CSF GXM exo-polysaccharide (μg/mL)	38	4.78 (1.28–16.27)
CSF viscosity, ratio to H_2_O	15	1.11 (1.09–1.27)
Ex vivo CSF cryptococcal cell measurements		Median (Range)
Total cell diameter^a^, μm	122	16.1 (10.3–29.8)
Capsule size^a,b^, μm	122	5.3 (2.9–10.7)
Capsule as % of cell diameter	122	65.1 (52.3–77.4)
% cells budding	118	10 (1–46)
% large cells, total diameter >30 μm	122	0 (0–44)

Abbreviations: CFU,colony-forming units; CSF, cerebrospinal fluid; GXM, glucuronoxylomannan; IQR, interquartile range; LFA, lateral flow assay.

^a^ Values derived using intra-patient mean diameters, from measuring 50 yeasts per patient CSF sample.

^b^ Capsule size = (total diameter-cell body diameter)/2.

Using intra-subject mean capsule size, the median (IQR) ex vivo capsular size for the cohort was 5.3 (4.6–6.3) μm, with capsule comprising almost two-thirds (65%) of total cell diameter. Quantification of baseline CSF GXM and GXM shedding of its corresponding *C. neoformans* isolate in vitro was performed on the first 38 participants. In CSF, median (IQR) GXM concentration was 4.8 (1.28–16.27) μg/mL. Using CrAg lateral flow assay (LFA) titers (n = 119) as a semi-quantitative measure of capsular shedding into CSF, mean capsule size correlated with CrAg shedding (*r* = 0.27, *P* = .005), including after adjustment for fungal burden (multivariable regression, *P* = 0.024).

CSF viscosity was measured in a total of 38 samples, of which 15 were at baseline. Median viscosity relative to water was 1.1. CSF viscosity in baseline and serial CSF samples correlated with fungal burden (*r* = 0.52, *P* < .001) and ex vivo GXM shedding (*r* = 0.72, *P* = .003) but not with CSF protein (*r* = 0.1, *P* = .6).

### Strains Producing Larger Cells and Capsules are Associated With Higher Opening Pressure

**Figure 1.**
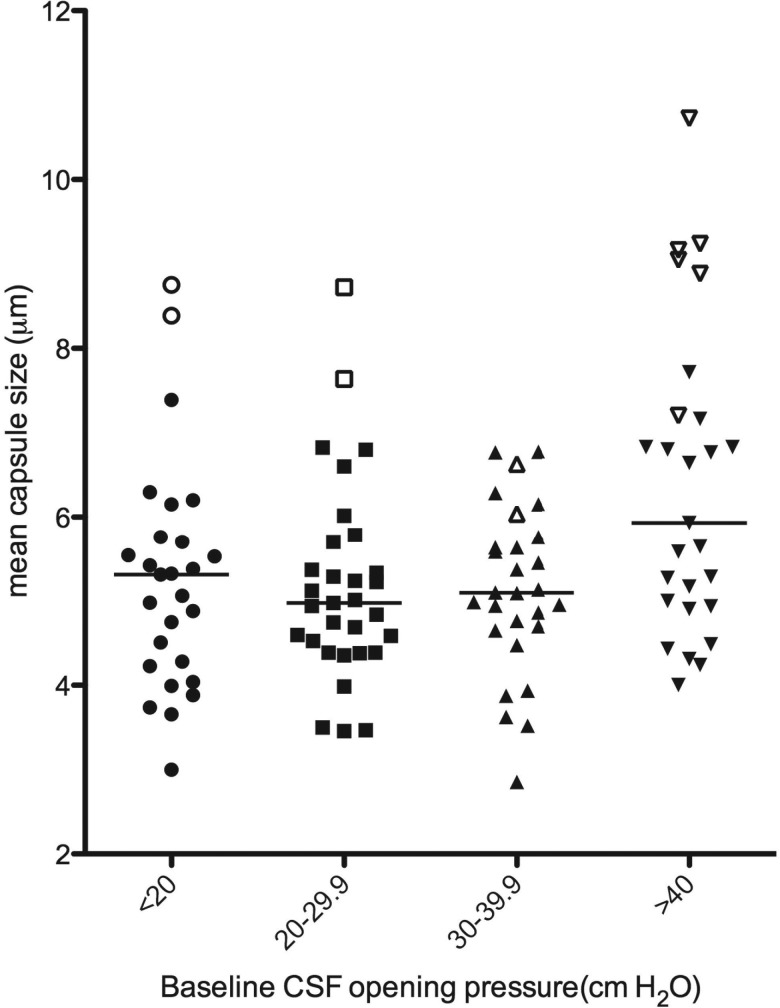
Dot plot of intraperson mean capsule size, stratified by CSF opening pressure category, n = 122. For the 4 opening pressure categories, median (IQR) ex vivo capsule sizes were as follows: <20 cm H_2_O: 5.3 (4.2,5.8) μm; 20–29.9 cm H_2_O: 5.0 (4.4,5.7) μm, 30–39.9 cm H_2_O: 5.1 (4.7,5.7) μm and >40 cm H_2_O: 5.9 (4.9,7.2) μm (Kruskal-Wallis *P* = .02). The 15 patients with large cells (>5 cells with total diameter >30 μm) are indicated by open symbols. Abbreviations: CSF, cerebrospinal fluid; IQR, interquartile range.

Average baseline CSF capsule size correlated with baseline CSF OP (*r* = 0.22, *P* = .02). By stratifying the cohort into OP categories, it became apparent that this effect was particularly driven by the patients with extremely high OP >40 cm H_2_O (Figure 1).

**Figure 2.**
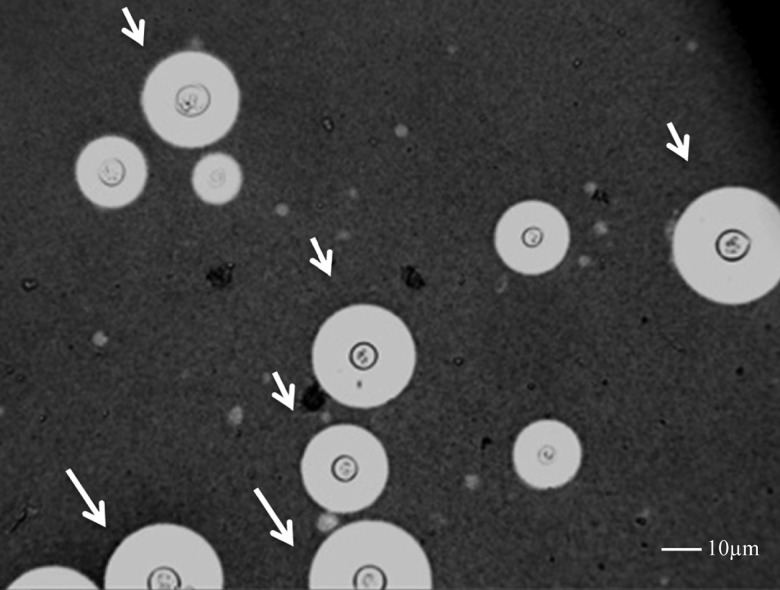
Microscopy of CSF (India Ink counterstain, ×40 objective), in a patient with CSF opening pressure > 55 cm H_2_O. White arrows indicate large cells (total diameter > 30 µm), ranging between 30.5 and 41.5 µm in diameter. Scale bar 10 µm. Abbreviation: CSF, cerebrospinal fluid.

The correlation between total cell diameter and baseline opening OP was of borderline significance (*r* = 0.18, *P* = .05). Although only 3.6% (441/12 197) of all the cryptococcal cells measured in human CSF in all subjects at all time points were large cells (total diameter >30 μm), 15 persons accounted for 80% (352/441) of all the large cells observed. In these subjects, large cells accounted for up to 62% of all cells measured. The cell enlargement was due to both capsular (mean size 8.1 μm vs 5.2 μm with and without large cells, *P* < .001) and cell body enlargement (mean diameter 8.2 μm vs 5.7 μm, *P* < 0.001) and thus not consistent with cryptococcal titan cells observed in mice (Figure 2) [22, 23]. Those with large cells had higher CSF CRAG titres compared to those without (median titers 1:14 400 vs 1:8000, *P* = .03). 13 of 14 with OP recorded had OP>30 cm H_2_O, and 9 of 14 (64%) suffered extremely high OP >40 cm H_2_O, compared to only 29% in those without large cells (*P* = .01; Figure 1, open forms indicate patients with large cells).

Using CrAg LFA titers, there was no correlation between ICP and CrAg shedding in CSF (*r* = − 0.04, *P* = .67, n = 112). In the much smaller subset of patients with CSF GXM and CSF viscosity measurements, there were no significant correlations between ICP and GXM shedding (*r* = − 0.05, *P* = .76, n = 37) nor CSF viscosity (*r* = 0.17, *P* = .58, n = 15). There were also no associations between ICP and in vitro capsule size (*P* = .59) or in vitro shedding (*P* = .31).

### Viscosity of GXM Exo-polysaccharide Does Not Differ Between Normal and High ICP Strains

**Figure 3.**
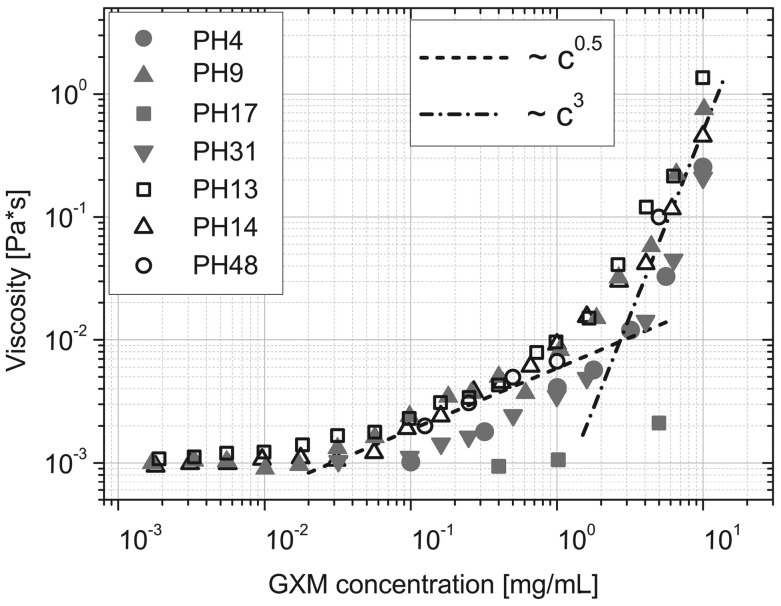
Log-log plot of viscosity (using optical tweezers) vs concentration of purified GXM exopolysaccharide dissolved in distilled water, from 7 clinical *Cryptococcus* *neoformans* strains [raised ICP (>20 cm H_2_O) filled symbols; normal ICP (≤20 cmH_2_O) open symbols, isolate ID in legend]. In all strains, the increase in viscosity with increasing concentration was non-linear, with viscosity increasing in proportion to the square root of concentration in the range of 0.02–2 mg/mL (gradient c^0.5^), and in proportion to the cube of the concentration in the range of 2–10 mg/mL (c^3^), typical of rod-like macromolecules in concentrated solutions [23]. Abbreviations: GXM, glucuronoxylomannan; ICP, intracranial pressure.

Figure 3 displays the viscosity of purified GXM exo-polysaccharide isolated from in vitro culture and diluted in water across the concentration range 1 μg/mL–10 mg/mL, for 7 clinical *C. neoformans* isolates, 4 of which were from persons with raised ICP (>20 cm H_2_O, filled symbols) and 3 with normal ICP (open symbols). At physiologic concentrations equivalent to those found in CSF (10^−3^ mg/mL), high and normal ICP strains showed no differences in GXM viscosity.

### Strains Producing Larger Capsules are Associated With Paucity of CSF Inflammation and Slower Rate of Clearance From CSF

**Figure 4.**
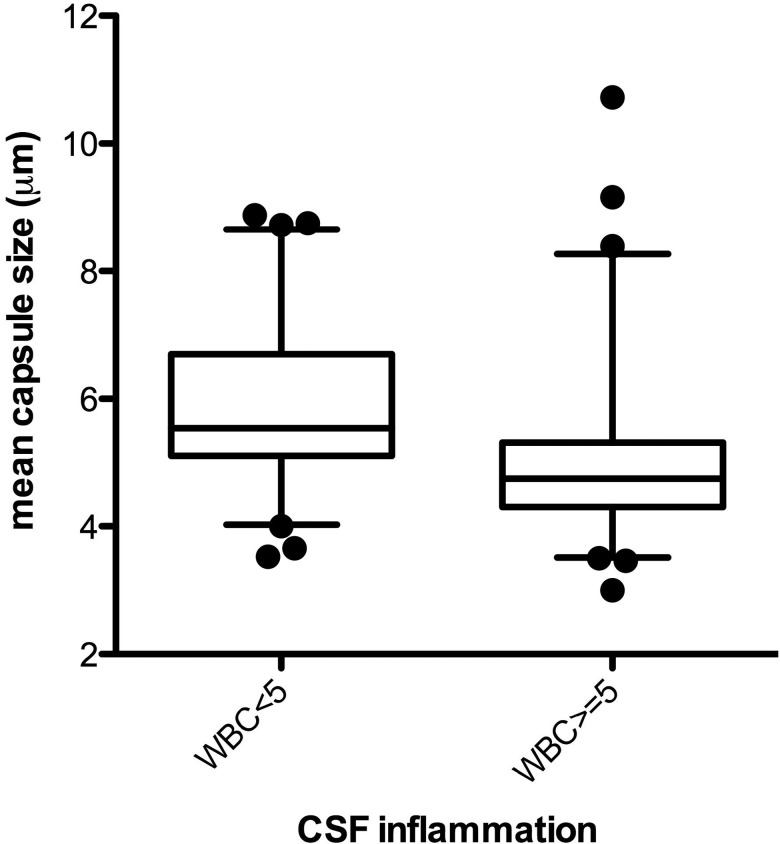
Box plot (median, IQR, 5–95th percentile) of ex vivo mean capsule size, stratified by absence or presence of CSF inflammation (WBC ≥5 cells/μL), n = 115, *P* < .001. Persons without a CSF WBC pleocytosis had *Cryptococcus* with larger capsules in CSF. Abbreviations: CSF, cerebrospinal fluid; IQR, interquartile range; WBC, white blood cell.

Average ex vivo capsule size inversely correlated with CSF WBC count (*r* = − 0.33, *P* < .001) and the cytokines IFN-γ (*r* = − 0.21, *P* = .03), IL-4 (*r* = − 0.23, *P* = .02), IL-6 (*r* = − 0.24, *P* = .01), IL-7 (*r* = − 0.20, *P* = .04), and IL-8 (*r* = − 0.22, *P* = .03). Figure 4 displays the distribution of capsule sizes stratified by presence (WBC ≥5 cells/μL, n = 54) or lack (WBC <5 cells/μL, n = 61) of CSF inflammation, with a larger ex vivo capsule being associated with paucity of inflammation (median 4.9 μm among WBC≥5/μL vs 5.5 μm among WBC<5/μL, *P* < .001). This was also true for total diameter (median, 15.3 μm vs 17.0 μm; *P* < .001), largely driven by the increased capsule size.

An increase in capsule size was associated with a slower rate of clearance of infection, adjusted for baseline fungal burden. The EFA for the cohort was −0.34 log_10_ CFU/mL CSF/day. For every 1 μm increase in mean capsule size, the EFA decreased by 0.04 CFU/mL CSF/day (SE 0.02, *P* = .04). In prior studies, higher levels of IFN-γ were associated with more rapid clearance of infection [24, 25]. This was not the case in this cohort, and adjustment for IFN-γ did not alter the association of capsule size with EFA (coefficient, 0.045; SE = 0.02, *P* = .04).

There was no significant association of capsule size with CD4 count (*P* = .59), altered mental status (*P* = .37) or 2-week mortality (*P* = .88).

### Intracranial Pressure Remained Associated With Capsular Size on Multivariable Analysis

On univariate linear regression, CSF parameters significantly associated (*P*≤.05) with opening pressure were mean capsule size, CSF protein, TNF-α, IFN-γ, IL-6, and IL-8. A multivariable regression model predicting OP was run using data from 97 patients with complete data for all of these parameters; CSF fungal burden was also included. Mean capsule size remained independently associated with CSF OP (β = 2.4, 95% CI: .5–4.2, *P* = .01) (Table 2).

**Table 2.** CSF Parameters Associated With CSF Opening Pressure on Univariate and Multivariable Regression Analysis

	Univariate Regression			Multivariable Regression		
Covariate	Coefficient^a^	95% CI	*P* Value	Coefficient^a^	95% CI	*P* Value
Average capsule size (μm)	2.50	.78, 4.22	.01	2.36	.51, 4.22	.01
Protein (mg/dL)	−0.02	−.04, −.003	.02	−0.02	−0.04, .002	.07
TNF-α (log_2_ pg/mL)	−2.29	−4.28, −.29	.03	−2.15	−5.22, .91	.17
IFN-γ (log_2_ pg/mL)	−1.51	−3.05, .02	.05	0.55	−1.83, 2.92	.65
IL-6 (log_2_ pg/mL)	−1.03	−2.00, −.07	.04	0.75	−1.06, 2.57	.41
IL-8 (log_2_ pg/mL)	−2.08	−3.80, −.36	.02	−1.5	−4.62, 1.63	.34
Fungal Burden (log_10_ CFU/mL)	0.50	−1.98, 2.98	.69	−0.48	−2.94, 1.98	.70

Abbreviations: CI, confidence interval; CFU, colony-forming units; CSF, cerebrospinal fluid; IFN, interferon; IL, interleukin; TNF, tumor necrosis factor.

^a^ Linear regression coefficient is the average change in CSF opening pressure (cm H_2_O) per 1 unit change in the covariate.

### Cell and Capsule Parameters Remained Constant Over 2 Weeks' Antifungal Treatment

Mixed effects models of change in parameters over time were analyzed among 74 persons with >2 observations over 2 weeks of antifungal therapy. There were no significant changes in capsular size (β = − 0.01, *P* = .41), cell body diameter (β = − 0.0, *P* = .97), or proportion of large cells (β = 0.0, *P* = .99) over time.

Variables showing significant decrease over time, in addition to fungal burden (mean change −0.31 log_10_ CFU/mL CSF/day, *P* < .001), were CSF OP (mean change −0.62 cmH_2_O per day, *P* = .002), CSF WBC count (−3.4 cells/µL/day, *P* = .004) and CSF protein (−0.40 mg/dL per day, *P* < .001).

### Ex Vivo Capsular Phenotype is not Reproducible In Vitro

Induced capsule size in vitro was measured for 58 serial isolates from 41 patients and compared to ex vivo measurements from corresponding CSF samples. There was no correlation between ex vivo and in vitro capsular size (*r* = − 0.11, *P* = .4). Mean capsular diameters were larger and more heterogeneous ex vivo than following in vitro culture in capsule-inducing media, median (range) 4.7 (0.9–9.5) vs 2.0 (1.1–4.3) μm, respectively. In addition, no correlation was observed between ex vivo and in vitro GXM shedding (*r* = − 0.16, *P* = .49), even after adjustment for fungal burden in CSF (*r* = − 0.22, *P* = .5).

## DISCUSSION

To our knowledge, this is the first study to examine CSF capsular phenotype in human cryptococcal meningitis and its relationship with host clinical and immune parameters. We found that persons infected with *C. neoformans* strains that produced larger capsules were significantly more likely to have raised ICP at presentation, less CSF inflammation, and slower rates of fungal clearance during treatment with amphotericin B and fluconazole.

**Figure 5.**
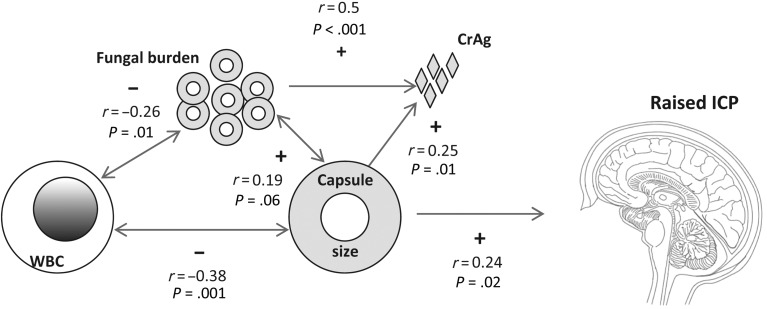
Schema of proposed inter-relationships between CSF fungal burden, capsule size, CrAg, CSF inflammation (WBC count) and raised intracranial pressure (Correlations shown as positive (+) or negative (−) by Spearman ρ). The arrows show possible directionality of these associations, which interact to produce mechanical obstruction and raised ICP. An example might be a highly encapsulated strain that elicits less CSF inflammation and thus also replicates more effectively, producing higher CSF fungal burden and antigen shedding. The combination of these factors results in raised ICP. Abbreviations: CrAg, cryptococcal antigen shedding; CSF, cerebrospinal fluid; ICP, intracranial pressure; WBC, white blood cell.

We have previously shown a relationship between high fungal burden and raised ICP, such that high fungal burden appeared necessary but not sufficient to produce raised ICP [10]. Although in this cohort fungal burden was not associated with ICP, it is notable that strains with higher fungal burden at diagnosis tended to have larger capsular size (*r* = 0.19, *P* = .06). Based on these findings, the relationship between capsule size, paucity of CSF inflammation, fungal burden, and raised ICP seems to be one of inter-related variables with multidirectional potential interactions, resulting in a particular clinical presentation (Figure 5).

Average capsular size remained associated with ICP on multivariable analysis, independent of the other factors. This supports the hypothesis that cryptococcal capsular phenotype directly influences the development of raised ICP in humans, as suggested in the rat ICP model [16]. This may simply be due to higher proportions of larger yeasts causing mechanical outflow obstruction in the context of a high fungal burden, supported by the observation that the 15 persons with highest proportions of large cells almost all developed high ICP.

Conversely, CSF inflammation did not appear to drive increased ICP: those with lower concentrations of pro-inflammatory cytokines in CSF had higher ICP. In contrast to CM in HIV-uninfected patients [26, 27], CM in HIV/AIDS is a pauci-inflammatory infection characterized by large fungal burdens. Cerebral edema is rare on postmortem [11, 28, and 29]. Additionally, corticosteroids, the treatment of cerebral edema, do not reduce ICP in AIDS-related CM [30].

Differences have been noted in the cell and capsule sizes of *Cryptococcus* isolated from diverse organs, such as lung and brain, observed in both human and mouse studies [31]. In this study, we necessarily assumed that capsular size in CSF is representative of that found in the brain and, specifically, the arachnoid villi. A small postmortem study demonstrated highly encapsulated cryptococci within arachnoid cells, whose numbers per high power field correlated with ICP [11]. The association between capsule size in arachnoid cells, fungal burden, and raised ICP in patients dying of CM needs to be explored in larger postmortem series.

Strains with larger capsules shed more CrAg, and CrAg titers correlate with CSF fungal burden [32]; however, CrAg shedding (by titer or GXM quantification) was not associated with raised ICP. CSF viscosity was also explored as a possible explanation for increased ICP. Although viscosity increased with fungal burden, the degree of change was small relative to viscosity of water. Although GXM exo-polysaccharide is viscous when dissolved in water at mg/mL concentrations [33], the conformation of GXM is relaxed and viscosity decreases in cationic solutions like CSF. At concentrations <50 µg/mL as found in rodents [16] and human CSF in our study, GXM is less likely to disrupt normal CSF mechanics across the arachnoid villi [9], unless the arachnoid channels act as filters, with GXM accumulation and polymerization occurring in situ.

GXM is structurally different between strains, which we attempted to study by comparing viscosity of purified culture supernatants. This revealed similar viscoelastic properties but few differences between strains, at a range of concentrations in water. Again, this may not be representative of the in vivo situation: the conformation of GXM exo-polysaccharide produced in vitro varies according to the isolation method and differs to that of bound capsule [34].

In vitro capsular size and shedding showed no correlation with that of the same strains ex vivo in human CSF, consistent with the lack of correlation found in mouse brains [14, 35]. In CSF from this African cohort, poorly encapsulated cells were rare, unlike some early reports in AIDS patients [36]. Cells and capsules were larger and far more heterogeneous than in vitro, consistent with a more evolved, chronic infection [37].

In addition to pathogen factors, there might be host factors contributing to development of raised ICP, for example, a difference in capacity for CSF absorption. This could be further elucidated using CSF outflow resistance measurement by lumbar infusion methods or phase-contrast magnetic resonance imaging (MRI), techniques that have been successfully applied in exploring other etiologies of communicating hydrocephalus [38, 39]. Beyond the arachnoid to dural sinus route, a second CSF outflow route is via lymphatics in perineural and perivascular channels [40]. In this regard it is interesting to note MRI brain findings of dilated Virchow-Robin spaces (perivascular channels ultimately draining into cervical lymph nodes), found to be full of fungi on postmortem, in 3 CM deaths [41].

Given the lack of in vitro-ex vivo correlations, our study illustrates the importance, as well as the challenges, of studying the relative contribution of virulence factors such as the cryptococcal capsule to clinical presentation in vivo, specifically in humans where, unlike in animal models, one is unable to standardize for host genetic and immune background, fungal inoculum, or timing of infection. Our finding of a significant independent association between capsular size and raised ICP, after adjustment for potential confounders, is thus likely to have pathophysiologic implications. To show causation, one would need to replicate these findings using a selection of highly encapsulated clinical strains, together with appropriate controls, in the rat ICP model.

In conclusion, we have demonstrated that phenotypic variation occurs in clinical cryptococcal strains, and that differences in the in vivo capsule phenotype of infecting *C. neoformans* strains have significant and important clinical associations. Strains producing larger capsules were associated with raised ICP as well as poorer CSF inflammatory response and slower rate of fungal clearance.
